# Mealworm (*Tenebrio molitor*): Potential and Challenges to Promote Circular Economy

**DOI:** 10.3390/ani11092568

**Published:** 2021-08-31

**Authors:** Roberta Moruzzo, Francesco Riccioli, Salomon Espinosa Diaz, Chiara Secci, Giulio Poli, Simone Mancini

**Affiliations:** 1 Department of Veterinary Sciences, University of Pisa, Viale delle Piagge 2, 56124 Pisa, Italy; roberta.moruzzo@unipi.it (R.M.); salomon.espinosadiaz@phd.unipi.it (S.E.D.); chiara.secci@phd.unipi.it (C.S.); simone.mancini@unipi.it (S.M.); 2 Department of Pharmacy, University of Pisa, Via Bonanno 6, 56126 Pisa, Italy; giulio.poli@unipi.it

**Keywords:** feed, food, edible insect, sustainability, frass, biofuel, chitin, chitosan, pharmaceutical

## Abstract

**Simple Summary:**

The main objective of this review is to analyse the potential of insects from the perspective of circular economy, focusing our attention on mealworm larvae. After pointing out the key concepts of circular economy and describing the use of insects in bioconversion processes, we discuss the most relevant uses of the mealworm in different industries, which show the great contribution this insect can make within circular productive systems. This topic has attracted a lot of attention due to its implications from an economic and environmental point of view. Recently, mealworm larvae were positively assessed by European Food Safety Authority (EFSA) as a safe novel food. As a matter of fact, the mealworm is the first edible insect to achieve this important milestone in the EU. Due to this new scientific opinion, considerable expectations arise on mealworms and their potential in different fields, which will surely lead to market developments in the following years.

**Abstract:**

Over the last few years, the concept of Circular Economy (CE) has received a lot of attention due to its potential contribution to the Sustainable Development Goals (SDGs), especially by reconciling economic growth with the protection of the environment through its grow-make-use-restore approach. The use of insects in circular production systems has been a good example of this concept as insects can transform a wide range of organic waste and by-products into nutritious feedstuffs, which then go back into the production cycle. This paper explores the potential of mealworms (*Tenebrio molitor*) in circular production systems by reviewing their use and applicability in several industries such as pharmaceuticals, agriculture, food, etc. Despite the high versatility of this insect and its potential as a substitute source of nutrients and other valuable components, there are still many legislative and behavioural challenges that hinder its adoption and acceptance.

## 1. Introduction

The Circular Economy (CE) concept first appeared in the 1960s in Kenneth Boulding’s essay “The Economics of the Coming Spaceship Earth” [1] aiming to transform the linear pattern of production and consumption by adopting strategies of a circular or ‘‘closing-the-loop” system in industrial production systems [2]. If human activities did not require the current exploitation rates of natural resources in the past, today, the effects of human activities would exceed the resilience of ecosystems on a global scale. In fact, over the last decade, CE has become one of the most important topics worldwide [3,4]. CE has received significant attention on the political agenda because of its potential for economic growth in a sustainable way [5,6,7]. In particular, CE can contribute to the United Nations Sustainable Development Goals (SDGs), with a strong and direct environmental impact in key goals such as SDG7 (affordable and clean energy) and SDG12 (responsible consumption and production), as well as in goals oriented to the economic dimension such as SDG8 (decent work and economic growth) [8]. In contrast with the linear economy (take-make-use-dispose) [9], the circular economy (grow-make-use-restore) [10] is a model that “aims to maintain components, materials, and products at their highest utility to eliminate waste from a system” [5]. In effect, CE envisions a future in which the concept of “waste” is phased out. In this new economic system, the waste needs to be transformed into biological and technical “nutrients” capable of satisfying the needs of human societies [11]. As reported by Cerdá and Khalilova [12] the CE key principles are lower inputs and lower use of natural resources; shared energy and priority to renewable and recyclable resources; reduction of emissions; reduction of material resource losses and wastage; upkeeping component quality and using inexpensive materials. Although academics and practitioners widely use the concept of CE, its meaning is still debated [13,14,15].

Based on an analysis of 114 definitions in the literature, throughout this paper, we decided to use the definition of Kirchherr et al. [16], who defined in their study: “CE is an economic system that replaces the ‘end-of-life’ concept with reducing, alternatively reusing, recycling and recovering materials in production/distribution and consumption processes”. 

**Figure 1:**
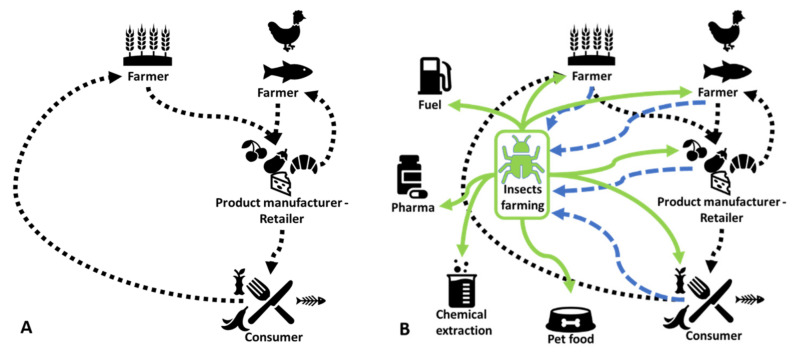
Enhancing of Circular Economy via insects farming. (**A**) and (**B**) represents the strengthening of the circular economy via edible insect farming.

Furthermore, recent studies have shown interest in the role of insects in circular food systems [2,17,18]. Derler et al. [19] stated that there are some reasons that justify the increased attention to insects in the circular economy (CE): insects can address the food waste and food loss problem, thanks to their capacity to convert organic matter into protein. Insect rearing involves less space, less water, and often also less energy compared with other conventional livestock. Insects can also contribute to more balanced human and animal diets thanks to their rich nutrient profile. Insects serve as an alternative source of nutrients and other substances by efficiently transforming organic residues and manure into nutritious biomass. The by-products derived from their production, such as the insect frass, can be used as a fertiliser. This enables the reintroduction of insect rearing substrates back into the food production chain, which is consistent with the circular economy’s principles [20] and SDGs [21] (Figure 1). The importance of insects in CE was pointed out by Cadinu et al. [22]. These authors provided a short review on the circularity of insect rearing and argued that insect farming was an advantageous choice within CE.

Insects could be used both as feed and food. Both uses make insects lawfully a kind of “livestock”; therefore, all the regulations regarding animal feed, husbandry, health, welfare and hygiene must be applied. Insects are a promising and more sustainable alternative to conventional protein feed, such as plant and fish meals, due to their low environmental impact and ability to enhance organic waste, even if their price in the EU market is not competitive yet [23]. 

The specific aim of this article is to acquire knowledge on the link between insects and the circular economy, analysing the mealworm (*Tenebrio molitor* L. 1758; Coleoptera, Tenebrionidae), one of the most promising insect species for human and animal consumption. After introducing the potential use of mealworms to upgrade food waste, this review presents some research studies on the different use of this species of insect. The current status of mealworm processing and its importance in the circular economy is also discussed in detail. 

## 2. Mealworm Characteristics

The mealworm is a holometabolic insect (complete metamorphosis, four life stages as egg, larva, pupa and adult) that probably originated in the Mediterranean, and nowadays, it is distributed worldwide due to colonisation and trade [24]. Mealworms were historically considered a pest that affected stored grains (molitor meaning “miller” in Latin). However, in the last decades, they were intensively studied for feed-food purposes and waste management. Their larvae are characterised by a rich nutritional profile, with an average of 50% (on dry matter-DM) of crude protein and about 30% (on DM) of crude fat [25,26], which may vary depending on the rearing substrates (fats more affected than proteins) [25,27]. Mealworm larvae have a well-balanced amino acids profile, rich in both essential and non-essential [28,29]. They are also a good source of fatty acids, with saturated fatty acids [30] characterised by myristic, palmitic and stearic acids [31]. In addition, the total amount of monounsaturated fatty acids and polyunsaturated fatty acids [29,32,33] is distinguished by a high content of oleic, linoleic, and linolenic acids [31] (Table 1). Moreover, mealworm larvae show a good composition in minerals and vitamins such as copper, iron, zinc, magnesium, potassium and phosphorus, [28,29] (Table 1), and vitamins E, B12, B3, B2, B5, and H [34].

Mealworm’s production has a small environmental impact, due to the few resources required for their rearing [19]. The amount of land required to obtain 1 kg of edible mealworm protein and the greenhouse gas produced is lower than that of chicken, cattle, and pigs [36]. Moreover, the water footprint per edible tons (m^3^/t) associated with mealworm production is comparable to chicken meat and lower than pig and beef meats [37]. They can be reared in a wide range of substrates and by-products derived from the food industry, which turn them into a great bioconversion tool, reducing food losses and fitting into the concept of sustainable CE [38]. In the last years, several substrates (insect feed) were tested in mealworm rearing, highlighting the insect’s certain plasticity [25,27,39,40,41]. At the same time, several different products have been obtained from mealworm rearing, and probably several more will be released in the next years. Despite mealworm potentiality, some restrictions could be represented by current legislation (Table 2). Still, things are changing fast, even in law regulations.

On the other hand, mealworms could also be related to some antinutritional aspects. The main factor affecting this negative characteristic is related to the presence of chitin, an insoluble fibre not expected to be digested in the small intestine of humans and several animals. Certainly, the degree of insect assumption must be considered to better understand the real effect on this antinutritional factor. Noteworthy, chitin, as several other fibres, could also affect the bioavailability of minerals and, therefore, play an indirect role in worsening the nutritional value of feed and food.

## 3. Use of Mealworm as Feed

The mealworm is one of the most attractive insect species in the search for alternative and sustainable feed sources. The use of insects as feed in animal farming is subject to several laws. The Commission Regulation (EU) 2017/893 authorised the use of processed animal protein (PAP) derived from seven insect species (*Hermetia illucens*, *Musca domestica*, *Tenebrio molitor*, *Alphitobius diaperinus*, *Acheta domesticus*, *Gryllus assimilis* and *Gryllodes sigillatus*) for feed in aquaculture systems. No authorisation is yet available for the use of insect PAPs in other livestock such as ruminants due to the restrictions implemented after the Transmissible Spongiform Encephalopathies (TSEs) outbreaks at the beginning of 2000 (Reg (EC) 999/2001). The authorisation extension to use insect PAPs as feed for other monogastric farm animals (poultry and swine) was recently positively evaluated by the EU authorities. By contrast, fats derived from insects (insect oil) can be used as feed in aquaculture and monogastric animals. In addition, dried or frozen whole insects (not milled, as reported in regulation (EU) 1017/2017 based on regulations (EU) 68/2013 and 1069/2009) are allowed for livestock, while the use of live insects as feed is allowed based on the national legislation in few EU member states. Due to their rich content in protein and energy, their larvae can be used as an ingredient for other feedstuffs or as a whole meal. Mealworms larvae may be used live, but also in the form of meals and oils, as a partial replacement of some conventional ingredients (soy-fish-maize-wheat meal/oil) [42,43,44]. Several authors analysed the effect of including mealworms meals/oils in livestock feeds in order to evaluate the potential utilisation of mealworms as feed and their effect on livestock growth performance, animal health and meat quality. Several authors reported the feasibility of replacing fishmeal with mealworm meal in aquaculture productions, especially in inclusion rates up to 25% [45,46]. Mealworm-based feeds were positively assessed for: rainbow trout (*Oncorhynchus mykiss*) [47,48], gilthead sea bream (*Sparus aurata*) [49,50], tench (*Tinca tinca*) [49], European sea bass (*Dicentrarchus labrax*) [51,52], blackspot sea bream (*Pagellus bogaraveo*) [53], tilapia (*Oreochromis niloticus*) [54,55] and Pacific white shrimp (*Litopenaeus vannamei)* [56,57].

Similarly, mealworm meal/oil were suitable alternatives to soybean meal/oil also for poultry production, again, when included in low amounts [26,44]. Different poultry production systems have been studied, such as broiler chickens [58,59,60,61], free-range chickens [62], Japanese quails (*Coturnix japonica*) [63] and Barbary partridge (*Alectoris barbara*) [64].

A few studies involve swine fed with mealworm meal as a partial or total replacement of soybean or fishmeal [26]. Results of feeding trials reported that the performance of weaning pigs [65,66] and growing pigs [67] was not affected by insect meal inclusion. Mealworms oil [68] and meal [69] were also suitable ingredients in rabbit feeds, even though more studies are needed to better understand the antimicrobial activity and impact on the gut microbiota of insect meals in this animal species [70]. Indeed, some studies showed that the chitin present in mealworm larvae could affect animal immune system traits, and even improve disease resistance and enhance beneficial gut microbiota [71]. Studies about the use of mealworms as feed on ruminants are lacking or have not been fully investigated due to the risk of TSEs. 

## 4. Use of Mealworm as Food

Due to their high nutritional value and the sustainability of their production, insects could also be a viable solution to meet the rising food demand for human consumption [72]. The Regulation (EU) 2015/2283 of the European Parliament and the Council of 25 November 2015 classified “whole insects and their parts” as food categories included in Novel Food. The use of insects for human consumption is currently allowed in a few EU member states according to article 35.2 of Regulation (EU) 2015/2283. This article provides transitional measures to allow foods that do not fall within the scope of Regulation (EC) No 258/97 (such as insect-based products in several EU countries), but which were legally placed on the market before 1 January 2018 (date of enforcement of Regulation (EU) 2015/2283), so that they can continue to be placed on the market for a certain period and under specific conditions. The commercialisation of insects on the market has not been fully enabled, as a matter of fact, up to now only two insect species have been included in the union list of novel foods authorised. 

The mealworm is the first insect species to receive a positive opinion from the European Food Safety Authority (EFSA) as novel food [73]. According to the evaluation carried out by EFSA, the whole insect larvae, thermally dried (blanched or oven-dried) or in powder (dried and grounded larvae), or added to several food products such as snacks, pasta and biscuits, could be consumed by all population groups. Following EFSA’s opinion and after a positive vote of the Standing Committee on Plants, Animals, Food and Feed (Novel Food and Toxicological Safety section), on 3 May 2021, the Commission adopted the regulation, giving the “green light” for the placing on the European market of dried yellow mealworm (whole or in the form of powder), based on the submission of novel food application. During the writing of this review, a second positive opinion from EFSA was released about the request to use frozen and dried formulations from migratory locusts (*Locusta migratoria*) as a novel food. This second positive opinion strengthens the attention of the European community about the insect sector and its future prospects.

Several research studies have evaluated the use of mealworms as an ingredient in well-known foods such as bakery products (e.g., bread, biscuit and snack) and protein bars.

The use of several cooking techniques (boiling, frying, vacuum and oven cooking) influences mealworms nutritional quality, with different effects on reducing the microbiological load and preserving their nutritional value [74,75]. The better results are obtained with boiling and vacuum cooking, ensuring product safety without altering the composition of macronutrients [74,75].

In addition, different drying methods (rack oven drying, vacuum drying and freeze-drying) could modify larvae colour and volatile compound profiles related to Maillard reactions [76], as well as blanching, microwave drying, freeze-drying, and combined treatments affect the mealworms colour and nutritional quality [77]. Additionally, the industrial processes involved in the production of mealworm powders may affect certain aspects of the final product, such as the physical and physicochemical properties, colour and morphological characteristics, which lead to different perceptions about appearance, flavour, texture, and overall acceptance [78].

Moreover, defatted mealworm powder showed high antioxidant capacities and could be used in food production as a functional ingredient [79]. Similarly, Zielìnska and Pankiewicz [80] reported that shortcake biscuits enriched with mealworm powder provide a high nutritional value and health-promoting effects due to increased protein content, antioxidant capacity and slowly digested starch (slow rise of glucose blood level after digestion). 

Roncolini et al. [81] reported that the addition of mealworm powder (5–10%) as a fortification component of bread improved the bread’s softness, volume, protein, and amino acids content. However, when compared to powders from other insects, such as house cricket (*Acheta domesticus*), mealworm powder showed the worst rheological and technological properties in relation to breadmaking and did not improve bread characteristics (bread volume, crumb density, and moisture) [82,83]. Anyhow, insect-containing bread showed a higher protein content than the conventional one, leading to an enriched bread [83]. However, consumer acceptance of fortified bread compared to that of the common one was negatively affected by insect inclusion [81]. Future research should be conducted to better modulate insect powder characteristics to obtain nutritionally balanced products with improved technological properties [83]. 

Mealworm powder also showed interesting results when included as an ingredient in extrudates (base for ready-to-eat snacks) by providing higher values of protein and minerals. Therefore, it contributes to the production of healthier snacks, even if they are more compact and harder than other extrudates analysed [84]. A 10% of mealworm powder was the optimum quantity to enhance the nutritional profile to a level that allows the use of the statement “source of protein” on the label (Regulation (EC) No 1924/2006) while maintaining the structural characteristics (e.g., pores side, pore wall thickness distribution and porosity), the texture properties, and the digestibility of high-quality extruded snacks [85]. Mealworms could also be an alternative and viable ingredient to snack production with the 3D printed food technique [86].

Consumers acceptance of insect-based novel food is strictly related to familiarity, neophobia, product preparation and insect visibility [87]. When evaluating the suitability of mealworms as food, in tests involving sensory-liking, willingness to try and willingness to buy, results have shown that familiar food preparations implementing the addition of mealworms in a non-visible way are better accepted than those in which they are visible. This perception is also affected when it comes to the flavour of carrier foods (savoury and sweet) enriched with mealworms, with a preference towards savoury, which highlights the relevance of an appropriate combination. Consequently, insect-based food acceptability is strictly linked to aspects related to food presentation and ingredient combination [87].

In line with these findings, the study by Bartkowicz [88] showed the acceptance of bars with visible mealworms was lower than bars without insects or with grounded mealworms. Other aspects such as familiarity (previous knowledge or experience with entomophagy) [89] and also cultural factors (use of insects as food) influenced the judgment of consumers on insect-based foods [90]. More information is required about this species’ allergenicity because mealworms (as other insects) might induce allergy in people already allergic to crustaceans and dust mites [73,91].

## 5. Use of Mealworm Frass

Frass is the generic term that refers to insect larvae’ excrements or the mix of them with the rearing substrate. Usually, the exuviae are also considered part of the insect frass. 

The frass derived from the production of mealworm larvae can be utilised in different ways. It can be used as an organic fertiliser due to plants’ nutritional content and rapid assimilation [92,93]. Several authors have analysed the potential application of mealworm frass as a fertiliser. Poveda et al. [94] tested the influence of mealworm frass on plant growth (chard plants, *Beta vulgaris* var. *cicla*) and abiotic stress resistance (bean plants, *Phaseolus vulgaris*), revealing positive effects related to frass utilisation. Houben et al. [95] investigated the characterisation, mineralisation and microbial metabolic activity of mealworm on the soil properties and growth and nutrient uptake by barley (*Hordeum vulgare*). Interestingly, Houben et al. reported that frass could be used as a partial or complete substitution of mineral fertiliser (NPK, nitrogen-phosphorus-potassium), mostly in a context where the availability of mineral fertilisers is limited. Wu et al. [96] reported that mealworm frass could significantly improve the flower quality of marigold, prolong the flowering period and decrease lodging throughout the reproductive process. Nevertheless, further studies are required to establish the correct timing of when the biofertiliser should be applied and the specific amount of fertiliser that would achieve optimal results.

The frass obtained by mealworms can also be an excellent raw material for producing high-efficiency biochar for heavy metal removal in wastewater treatment or soil remediation [97]. Biochars made from this kind of frass performed better than biochar made from original crop residues. 

## 6. Use of Mealworm for Biodiesel Production

Biodiesel is a non-fossil fuel with a high cost and long-term impact on food prices due to oilseed dependency and arable land requirement [98,99]. To reduce the cost of biodiesel, researchers are studying alternative resources with considerably lower costs that could be used for biodiesel production, such as insects. The fat from some insects was proven to be a sustainable feedstock for biodiesel production [100,101,102,103]. In particular, insects can accumulate saturated fatty acids (i.e., C18 and C16) with physical and chemical properties, such as kinematic viscosity, calorific value, oxidation stability, conducive to further conversion into biodiesel [104]. Although there are different kinds of insects, few species have been studied to convert organic wastes into biodiesel [105].

To date, mealworm has only been investigated as a potential substitute for oilseeds by a few authors [100,106], analysing the potential use on different substrates for anaerobic digestion. Wang et al. [107] employed an innovative and environmentally friendly technology consisting of the application of *Tenebrio molitor* and *Hermetia illucens* to improve the corn stover utilisation to produce biodiesel, defatted larval meal, and biofertiliser. 

## 7. Use of Mealworm as a Source of Chitin and Chitosan

Chitin, the second most abundant biopolymer in nature after cellulose [108], and especially its deacetylated derivative chitosan, have attracted major scientific and industrial interest for its application in a wide range of fields such as agriculture, biotechnology, pharmaceuticals, medicine, wastewater treatment and more [109,110,111]. The great applicability of these materials is due to their biodegradability, antimicrobial effects, adsorption capacity and other intrinsic functional properties [112]. Currently, the main natural sources of chitin are crab and shrimp shells [113]. Still, it is argued that their availability is highly dependent on seasonality and the sustainability of their production is questionable [114]. Taking into consideration that the global market for chitin and chitosan is expected to increase with a compound annual growth rate of 15.4% [115], it is important to find new sources in order to satisfy this growing demand in a stable and sustainable way. One of the most attractive alternatives is the use of insects, and especially the use of mealworms. The extraction of chitin and chitosan from insects has proved to be more advantageous than existing sources in terms of extraction methods, chemical consumption, time and yield [116]. The study by Song et al. [117] indicated that the exuvium and whole body of mealworm larvae might serve as a source of chitin and chitosan for use in domestic animal feed. Son et al. [118] found that mealworm chitin shows a significantly softer texture than crustacean chitin with superior anti-inflammatory effects. Also, Shin et al. [119] found that chitosan from the mealworm has a similar structure as those of commercial chitosan and showed inhibition properties in several antimicrobial activity tests. 

## 8. Use of Mealworm as a Source of Bioactive Extracts and Compounds

Since mealworm has been recognised as a novel food, the scientific community’s interest in evaluating the presence therein of potential biologically active compounds, which may either enrich human diet or be used as functional ingredients into supplements and nutraceutical preparations, has grown exponentially. A considerable number of studies aimed at assessing the biological effect and even the therapeutic potential of different mealworm extracts has been recently reported in the literature, some of which identify specific bioactive substances responsible for potentially beneficial effects against pathological conditions.

The presence in mealworm larvae of bioactive peptides with antimicrobial activity, which are indispensable for the innate immunity of the insect, is well known. For instance, one of the first antimicrobial peptides isolated from the larvae haemolymph, tenecin 1, was identified about 25 years ago by Lee and co-workers and was found to inhibit the growth of Gram-positive bacteria [120]. In the following years, the same research group characterised three other peptides called tenecin 2–4 with antimicrobial activity against fungi and Gram-negative bacteria [121,122,123]. More recent studies focused on evaluating the biological activity of protein hydrolysates obtained from mealworm larvae suggest that this edible insect may represent a source of bioactive peptides endowed with specific enzyme inhibition activities that may be helpful against various disorders. Protein hydrolysates obtained using the Alkalase enzyme showed considerable activity against rabbit angiotensin-I converting enzyme (ACE). They determined a significant reduction of systolic blood pressure in spontaneously hypertensive rats (SHR). A specific tripeptide isolated from these hydrolysates showed ACE-inhibitory activity [124]. Other small peptides recently isolated from different protein hydrolysates obtained under simulated gastrointestinal digestion and absorption conditions showed pig ACE-inhibitory activity [125]. Finally, a very recent in vivo study demonstrated that a diet enriched in defatted larvae of mealworm determined an antihypertensive effect in SHR, with a significant reduction in blood pressure, that could be ascribed to ACE inhibition, as well as cardio- and neuroprotective effects that may be due to antioxidant and anti-inflammatory activities. These results suggest that mealworms may help treat borderline blood pressure values in humans and may be employed as an active ingredient in functional food for the non-pharmacological treatment of pre-hypertension or mild hypertension [126].

Other peptide-based bioactive compounds with potential beneficial effects against cardiovascular diseases were recently isolated from mealworms. In particular, the diketopiperazine cyclo(L-Pro-L-Tyr) and N-acetyltyramine showed in vitro end ex vivo antithrombotic activities similar to that of rivaroxaban (an oral anticoagulant) [127]. These activities included increased blood clotting time, delayed thrombogenesis and thrombogenic time, and succeeded in effectively and concentration-dependently inhibiting ADP- and collagen-induced platelet aggregation. Moreover, both compounds showed anti-platelet aggregation and antithrombotic activity in a mouse model of arterial and pulmonary thrombosis [128].

The antioxidant and anti-inflammatory activity of mealworm larvae protein hydrolysates were also recently observed. After in vitro gastrointestinal digestion and absorption, the hydrolysates obtained by Zielińska et al. showed cyclooxygenase-2 (COX-2) inhibition and also good free-radical scavenging activity [129]. Moreover, the treatment process generally applied to edible insects (baking and boiling) proved not to be detrimental to the hydrolysates’ biological activity [125,129]. In contrast, the type of enzymatic treatment and degree of hydrolysis was found to significantly impact the hydrolysates and be optimisable for modulating their peptide composition and thus the desired bioactive properties [130]. Finally, a very recent study demonstrated that, due to their antioxidant activity, mealworm hydrolysates could exert a cellular hepatoprotective effect, attenuating the H_2_O_2_-induced cytotoxicity in AML12 mouse hepatocytes, and two different bioactive peptides could be identified from such hydrolysates [131].

Mealworm extracts may also be endowed with anti-adipogenic and anti-obesity effects. In 2017, ethanol extracts of the larvae inhibited adipogenesis through the AMP-activated protein kinase (AMPK) pathway by increasing the enzyme phosphorylation during pre-adipocyte differentiation. Moreover, a daily oral administration of larvae powder attenuated body weight gain in high-fat diet-induced obese mice, efficiently decreased hepatic steatosis and lipid droplet accumulation, and reduced the levels of alanine transaminase (ALT) and aspartate transaminase (AST) enzymes [132]. In a further in vivo study employing obese Zucker Rats, a rat model of hyperlipidaemia, a pronounced reduction of liver and plasma lipid concentration was obtained by replacing casein with mealworm meal as a source of protein [133]. Moreover, extracts from mealworm lyophilised powders, especially those obtained through pressurised liquid extraction, showed inhibitory activity of pancreatic lipase, the enzyme responsible for the digestion of dietary lipids, thereby providing additional evidence of the potential impact of the extracts in lipid metabolism [134].

## 9. Conclusions

Sustainably feeding the growing human population is essential, and edible insects could help. The application of circular economy concepts to the production of sustainable and renewable sources of protein, in particular with regards to algae, microalgae, fungi and insects, represents a challenge for the future of humanity. A redesign of the food production chain is required and, it will necessarily imply systematic reuse of by-products, co-products and agri-food waste, in accordance with legislation. The mealworm could contribute to reduce losses and increase circularity, while new products and benefits could arise. From food to feed, chemicals to fertilisers, mealworms could be bred to obtain a valuable product, sometimes even more than one. By farming the mealworm, entire production chains could increase outcomes lowering environmental impacts while satisfying the increasing demands of goods. Insect farming, specifically mealworm farming, could increase the circular economy both in developed and underdeveloped countries. Tailormade marketing strategies and improvements in consumer awareness will help the entire process.

## Figures and Tables

**Table 1:** Mealworm larvae nutritional characteristics.

Amino Acids Profile				Fatty Acids Profiles				Minerals			
Essential amino acids	mg/g DW [29]	g/kg DM [35]	mg/g protein [32]		% total FA [33]	% total FA [29]	% total FA [32]		mg/kg DW [29]	mg/100 g DM [32]	mg/kg [34]
Valine (Val)	18.91	38.32	39.7	SFA	30.01	20.99	25.32	Iron (Fe)	184.17	3.29	20.7
Leucine (Leu)	22.06	41.28	45.8	Lauric acid (C12:0)	0.15	0.32	0.21	Zinc (Zn)	98.64	11.2	49.5
Isoleucine (Ile)	13.10	27.56	21.4	Myristic acid (C14:0)	4.14	2.12	2.63	Potassium (K)	8914	835	3350
Phenylalanine (Phe)	13.09	20.48	16.1	Palmitic acid (C16:0)	21.36	17.24	18.0	Calcium (Ca)	319.6	41	156
Methionine (Met)	6.01	7.63	9.6	Stearic acid (C18:0)	4.00	0.69	3.84	Magnesium (Mg)	2333.1	304	620
Lysine (Lys)	15.81	32.61	26.7	MUFA	46.67	47.35	43.27	Sodium (Na)	437.1	57	225
Histidine (His)	8.37	18.68	16.1	Palmitoleic acid (C16:1)	1.64	1.94	2.07	Copper (Cu)	20.15	1.86	8.3
Tryptophan (Trp)	2.98	6.75	-	Oleic acid (C18:1)	44.52	43.77	40.86	Selenium (Se)	0.13	-	0.123
Threonine (Thr)	12.66	22.62	26.1	Eicosenoic acid (C20:1)	0.22	-	0.16	Manganese (Mn)	18.88	-	3.2
Non-essential amino acids				PUFA	18.79	31.66	31.37	Chromium (Cr)	1.91	-	-
Cysteine (Cys)	11.86	5.58	5.5	n-6	18.23		29.68	Arsenic (As)	1.27	-	-
Taurine (Tau)	0.34	-	-	Linoleic acid (C18:2)	17.97	29.39	29.68	Cadmium (Cd)	0.08	-	-
Aspartic acid (Asp)	15.44	46.73	50.5	Arachidonic acid (C20:4n-6)	0.11	-	-	Palladium (Pd)	0.65	-	-
Serine (Ser)	13.61	26.74	28.8	n-3	0.56	-	1.61	Phosphorus (P)	-	-	2640
Glutamic acid (Glu)	39.19	65.83	79.7	Linolenic acid (C18:3)	0.33	2.27	1.61	Chloride (Cl)	-	-	1760
Glycine (Gly)	17.06	30.21	31.8	Eicosapentaenoic acid (C20:5n-3)	0.06	-	-	Iodine (I)	-	-	<0.10
Alanine (Ala)	24.83	41.16	44.3	Docosapentaenoic acid (C22:5n-3)	0.08	-	-				
Tyrosine (Tyr)	21.46	42.66	28.8	Docosahexaenoic acid (C22:6n-3)	0.09	-	-				
β-Alanine (β-Ala)	2.68	-	-	n-6/n-3	41.41	12.98	18.44				
Arginine (Arg)	18.85	30.67	25.6								
Proline (Pro)	20.01	38.30	43.4								

DW: dry weight. DM: dry matter. FA: fatty acid.

**Table 2:** Main inputs and outputs of insect farming sector in the European Union.

Input	By-products, processing waste	Vegetal, dairy, eggs and honey
		Meat and fish
	Former Foodstuff	Vegetal, dairy, eggs and honey
		Meat and fish
	Consumer food waste	Not allowed
	Slaughterhouse products	Not allowed
	Manure	Not allowed
Output	Animal feed	Insects’ PAP for aquaculture and pet
		Insects’ PAP for poultry and pigs
		Insects’ PAP for ruminants
		Insects’ fat for aquaculture, monogastric animals and pet
		Live insect ^1^ for aquaculture, monogastric animals and pet
		Whole insects (dried or frozen, not milled) for livestock (not ruminants)
	Food	Allowed in few EU member states ^2^
		Dried yellow mealworm (whole or powder) ^3^
		Other insects
	Frass	Partially allowed
	Chemicals, pharmaceuticals, cosmetics	Allowed
	Biofuel	Allowed

Cell colour: green (allowed); orange (partially allowed or in evaluation); red (not allowed). ^1^ Live insect: allowed basing on national legislation in certain EU member states. ^2^ Due article 35.2 of Regulation (EU) 2015/2283. ^3^ First EFSA positive opinion about insects as a novel food.

## Data Availability

Data are available on request.
